# Supplementary material for: High-Fiber Diet and Crohn’s Disease: Systematic Review and Meta-Analysis
Source: Nutrients. 2023 Jul 12;15(14):3114. doi: 10.3390/nu15143114 (PMC10384554; doi:10.3390/nu15143114)
Supplement: Supplementary file 1 [file nutrients-15-03114-s001.zip › Table S1.pdf]

Table S1. JBI Critical Appraisal for Randomized Clinical Trials

	Heaton K, et al.				Lacerda J, et al.			
	Yes	No	Unclear	NA	Yes	No	Unclear	NA
Was true randomization used for assignment of participants to treatment groups?			x		x			
Was allocation to treatment groups concealed?	x				x			
Were treatment groups similar at the baseline?			x				x	
Were participants blind to treatment assignment?		x			x			
Were those delivering the treatment blind to treatment assignment?	x					x		
Were treatment groups treated identically other than the intervention of interest?	x				x			
Were outcome assessors blind to treatment assignment?			x			x		
Were outcomes measured in the same way for treatment groups?	x				x			
Were outcomes measured in a reliable way?	x				x			
Was follow up complete and if not, were differences between groups in terms of their follow up adequately described and analysed?	x				x			
Were participants analysed in the groups to which they were randomized?	x				x			
Was appropriate statistical analysis used?	x				x			
Was the trial design appropriate and any deviations from the standard RCT design (individual randomization, parallel groups) accounted for in the conduct and analysis of the trial?				x				x
Overall appraisal	Included				Included			

Abbreviations: NA, Not applicable. Maximum score: 13 points.
