# Supplementary material for: High-Fiber Diet and Crohn’s Disease: Systematic Review and Meta-Analysis
Source: Nutrients. 2023 Jul 12;15(14):3114. doi: 10.3390/nu15143114 (PMC10384554; doi:10.3390/nu15143114)
Supplement: Supplementary file 1 [file nutrients-15-03114-s001.zip › Table S2.pdf]

Table S2. JBI Critical Appraisal for Quasi-experimental Studies

	Chiba M, et al. [29]				Chiba M, et al. [30]				Chiba M, et al. [31]			
	Yes	No	Unclear	NA	Yes	No	Unclear	NA	Yes	No	Unclear	NA
Is it clear in the study what is the 'cause' and what is the 'effect' (i.e. there is no confusion about which variable comes first)?	x				x				x			
Were the participants included in any comparisons similar?	x					x			x			
Were the participants included in any comparisons receiving similar treatment/care, other than the exposure or intervention of interest?	x					x			x			
Was there a control group?		x				x				x		
Were there multiple measurements of the outcome both pre and post the intervention/exposure?	x				x				x			
Was follow up complete and if not, were differences between groups in terms of their follow up adequately described and analyzed?	x				x				x			
Were the outcomes of participants included in any comparisons measured in the same way?	x				x				x			
Were outcomes measured in a reliable way?	x				x				x			
Was appropriate statistical analysis used?	x				x				x			
Overall appraisal	Included				Included				Included			

Abbreviations: NA, Not applicable. Maximum score: 9 points.
