# Supplementary material for: High-Fiber Diet and Crohn’s Disease: Systematic Review and Meta-Analysis
Source: Nutrients. 2023 Jul 12;15(14):3114. doi: 10.3390/nu15143114 (PMC10384554; doi:10.3390/nu15143114)
Supplement: Supplementary file 1 [file nutrients-15-03114-s001.zip › Table S3.pdf]

Table S3. JBI Critical Appraisal for Cohort Studies

	Schreiner P, et al.				Dolovich C, et al.				Brotherton C, et al.			
	Yes	No	Unclear	NA	Yes	No	Unclear	NA	Yes	No	Unclear	NA
Were the two groups similar and recruited from the same population?	x				x				x			
Were the exposures measured similarly to assign people to both exposed and unexposed groups?	x				x				x			
Was the exposure measured in a valid and reliable way?	x				x				x			
Were confounding factors identified?		x			x				x			
Were strategies to deal with confounding factors stated?		x				x				x		
Were the groups/participants free of the outcome at the start of the study (or at the moment of exposure)?		x				x				x		
Were the outcomes measured in a valid and reliable way?	x				x				x			
Was the follow up time reported and sufficient to be long enough for outcomes to occur?	x				x						x	
Was follow up complete, and if not, were the reasons to loss to follow up described and explored?	x				x				x			
Were strategies to address incomplete follow up utilized?				x				x				x
Was appropriate statistical analysis used?	x				x				x			
Overall appraisal	Included				Included				Included			

Abbreviations: NA, Not applicable. Maximum score: 11 points.
