# Supplementary material for: High-Fiber Diet and Crohn’s Disease: Systematic Review and Meta-Analysis
Source: Nutrients. 2023 Jul 12;15(14):3114. doi: 10.3390/nu15143114 (PMC10384554; doi:10.3390/nu15143114)
Supplement: Supplementary file 1 [file nutrients-15-03114-s001.zip › Table S4.pdf]

Table S4. JBI Critical Appraisal for Cross-sectional Studies

	Tasson L, et al.				Mirmiran P, et al.				Opstelten J, et al.			
	Yes	No	Unclear	NA	Yes	No	Unclear	NA	Yes	No	Unclear	NA
Were the criteria for inclusion in the sample clearly defined?	x				x				x			
Were the study subjects and the setting described in detail?	x				x				x			
Was the exposure measured in a valid and reliable way?	x				x						x	
Were objective, standard criteria used for measurement of the condition?	x				x				x			
Were confounding factors identified?	x				x				x			
Were strategies to deal with confounding factors stated?	x					x				x		
Were the outcomes measured in a valid and reliable way?	x				x				x			
Was appropriate statistical analysis used?	x				x				x			
Overall appraisal	Included				Included				Included			

Abbreviations: NA, Not applicable. Maximum score: 8 points.
